# Epigenetics in Captivity: Restoring Wild Phenotypes in Captive‐Reared Salmonids

**DOI:** 10.1111/eva.70210

**Published:** 2026-03-12

**Authors:** Tia Attfield, Andrew Honsey, Amanda Ackiss, Andreas Luek, Brian Meagher, Hayley Nuetzel, Ilana Koch, Julien April, Kristy Wakeling, Kyle W. Wellband, Raphaël Bouchard, Sarah J. Lehnert, Shawn Narum, Timothy M. Healy, Trevor E. Pitcher, Clare J. Venney

**Affiliations:** ^1^ Department of Biological Sciences University of Alberta Edmonton Alberta Canada; ^2^ U.S. Geological Survey Great Lakes Science Center Ann Arbor Michigan USA; ^3^ Alberta Environment & Protected Areas Edmonton Alberta Canada; ^4^ Columbia River Inter‐Tribal Fish Commission Portland Oregon USA; ^5^ Ministère de l'Environnement, de la Lutte Contre les Changements Climatiques, de la Faune et des Parcs Québec City Quebec Canada; ^6^ Alberta Forestry & Parks Edmonton Alberta Canada; ^7^ Fisheries and Oceans Canada Pacific Sciences Enterprise Centre West Vancouver British Columbia Canada; ^8^ Université Laval Québec City Quebec Canada; ^9^ Fisheries and Oceans Canada Newfoundland and Labrador Branch St. John's Newfoundland and Labrador Canada; ^10^ Fisheries and Oceans Canada Pacific Biological Station Nanaimo British Columbia Canada; ^11^ Great Lakes Institute for Environmental Research University of Windsor Windsor Ontario Canada

**Keywords:** conservation ecology, DNA methylation, hatchery management, phenotype, salmonids, supplementation

## Abstract

Captive rearing is a common practice for the stocking, conservation, and supplementation of fish species worldwide, but captive‐reared fish can exhibit altered phenotypes leading to reduced fitness in nature compared to wild conspecifics. In salmonids, certain studies have found limited genetic differentiation between wild and captive‐reared fish. However, documented changes in gene expression in hatchery fish have led scientists to investigate epigenetic mechanisms, such as DNA methylation, as a source of these differences. In this binational collaborative piece, we synthesize the knowledge and efforts of academics and government scientists to highlight how interactions between captive rearing and the epigenome elicit parallel phenotypic changes across salmonid species. We examine the known and potential links between DNA methylation and the phenotypic effects of captive rearing including changes in behavior, color, gut microbiomes, and developmental abnormalities. We review efforts to minimize these phenotypic and epigenetic effects including attempts to modify the hatchery environment and rearing protocols. We provide a framework to integrate epigenetic considerations into hatchery rearing protocols by weighing the heritable nature of DNA methylation with the goals of different captive rearing programs and explore whether minimizing the phenotypic and epigenetic effects of captive rearing is worthwhile. We examine heritability and persistence of epigenetic effects, and we propose the exploitation of heritable bet‐hedging as an epigenetic buffer to increase post‐release survival. We also suggest novel applications of epigenomic biomarkers as a non‐lethal method for post‐release monitoring. Ultimately, collaborative multi‐disciplinary research across species is needed to understand the comprehensive effects of captive rearing, reduce the ecological impacts of captive fish in the wild, and increase population resilience. Integrating epigenetics into fish hatchery management will provide new opportunities for optimizing and improving captive rearing.

## 1 Introduction

### 1.1 Conservation Concerns

Aquatic systems are experiencing biodiversity loss at an unparalleled rate (Reid et al. 2019). In finfish—hereafter, “fish”—anthropogenic activities have contributed to the direct decline of populations due to pressure from harvesting large quantities of high‐value fish (Smith 1968; COSEWIC 2010a; Arkhipkin 2025), accidental catch of species under recovery (reviewed in Gray and Kennelly 2018), and discarding non‐target species as bycatch (Casey and Myers 1998; Harrington et al. 2005). Indeed, one of the most prominent threats to fish conservation remains the past and present overexploitation by fisheries (Neff et al. 2011). Still, humans have indirectly impacted freshwater and marine fish populations through habitat loss, environmental degradation and fragmentation, and invasive species introduction (reviewed in Meybeck 2004; Halpern et al. 2008; Su et al. 2021; WWF 2024). For migratory species that rely on freshwater and saltwater environments during their life cycle, populations are even more at risk (Deinet et al. 2024). In particular, anadromous salmonids face both marine and freshwater‐specific anthropogenic threats within their lifetime due to their migration across distinctly impacted landscapes. Salmonids are also frequently targeted commercially due to their high social, cultural, and steadily increasing economic value. This value, when combined with an extensive habitat range, may further complicate conservation approaches, increasing pressure on an already vulnerable group.

Additionally, salmonids may experience climate stress from rising water temperatures, and from the secondary effects of unpredictable weather patterns like drought (Singer et al. 2020), heat domes (Free et al. 2023), or alterations to stream flow dynamics (Mantua et al. 2010). Specifically, increasing temperatures may alter the timing of reproductive cues (e.g., reviewed in Pankhurst and Munday 2011) or intensify inter‐ and intraspecific competition between native and non‐native species (Van Zuiden et al. 2016; Cline et al. 2019; Jan et al. 2025), while erratic weather patterns can increase siltation, or alter habitat distribution via destruction and fragmentation of ecosystems. Anthropogenic structures used to prevent flooding, such as dams, roadways (e.g., culverts), or flow diversions, can likewise fragment ecosystems and disrupt the movement of salmon. Climate change is thus partially responsible for the current biodiversity crisis (Hollowed et al. 2013; Urban 2015), and presents a multidimensional threat to salmonids.

### 1.2 Captive Rearing: Fish Hatcheries

Captive rearing has been increasingly used to combat declines in finfish abundance and mitigate harm done to aquatic ecosystems. As of 2022, over 350 species of fish are currently being raised in captive environments worldwide (FAO 2024). Captive rearing for conservation purposes is also increasing. Many populations of salmonids are at risk, with many being considered endangered or threatened across the United States and Canada, including Chinook salmon ( *Oncorhynchus tshawytscha* ) (COSEWIC 2018; Endangered and Threatened Wildlife 2022) and sockeye salmon ( *Oncorhynchus nerka* ) (COSEWIC 2022a; Endangered and Threatened Wildlife 2022) on the Pacific coast, westslope cutthroat trout ( *Oncorhynchus clarkii lewisi* ) (Fisheries and Oceans Canada 2014) and shortnose cisco ( *Coregonus reighardi* ) (COSEWIC 2005) in central Canada, bull trout ( *Salvelinus confluentus* ) in central Canada and the United States (COSEWIC 2012; Endangered and Threatened Wildlife 2022), and Atlantic salmon ( *Salmo salar* ) (COSEWIC 2010b; Endangered and Threatened Wildlife 2022) and various *Coregonus* species (COSEWIC 2009, 2022b) in the eastern United States and Canada. Captive rearing has been employed to tackle the conservation concerns of salmonids, where these fish are then released into nature to supplement and enhance wild populations, albeit sometimes with unintended drawbacks.

Although conservation programs intend to supplement natural populations and, ideally, minimize divergence between hatchery and naturally produced fish, such programs often produce fish with reduced fitness in the wild including components such as survival and reproductive success (Araki et al. 2008; Christie et al. 2012; Bouchard 2021; Bouchard et al. 2022). Artificial environments can also cause physiological and morphological abnormalities (Poppe et al. 2003; Honsey, Anweiler, et al. 2024; Honsey, Kao, et al. 2024), precocious maturation at small body sizes (Larsen et al. 2004; Pearsons et al. 2009), and behavioral changes (Fleming and Gross 1993; Braithwaite and Salvanes 2005; Schroder et al. 2010). For example, decreased body size and irregular brain development are common in hatchery salmonids compared to wild conspecifics (Schroder et al. 2010; Wiper et al. 2014; Bokvist 2022). Particularly, hatchery environments are associated with reducing the size of several regions of the brain in rainbow trout ( *Oncorhynchus mykiss* ) (Marchetti and Nevitt 2003; Kihslinger and Nevitt 2006), whereas in Chinook salmon, hatchery fish exhibited larger brains relative to body size (Wiper et al. 2014). Variation in cardiac performance is also often reported across salmonids. This includes significant differences in heart morphology between farmed and wild Atlantic salmon and rainbow trout (Poppe et al. 2003), as well as diminished cardiac function during spawning in hatchery Chinook salmon (Twardek et al. 2021), both of which may impede migration abilities (Pedersen et al. 2008; Chittenden et al. 2010; Larsson et al. 2011). Hatchery environments can have inconsistent effects on behavior, sometimes leading to timidness and decreased dominance (Brockmark and Johnsson 2010; Schroder et al. 2010), and other times, increased boldness and aggression (Islam et al. 2020). Furthermore, some studies have documented that captive reared fish may reduce population fitness (Araki et al. 2007; O'Sullivan et al. 2020) and can lead to a reduction in effective population size (Ryman and Laikre 1991; Hindar et al. 1991), though protocols meant to curb domestication may mitigate these effects (Fast et al. 2015; Waters et al. 2015; Ford et al. 2016; Janowitz‐Koch et al. 2019).

### 1.3 Gene Expression, Transcription & Epigenetics

Captive rearing often has similar phenotypic and fitness effects across salmonid species (Koch and Narum 2021), but the molecular underpinnings of these parallel effects are not well understood. It is difficult to identify a singular cause of the phenotypic divergence seen in captive‐reared fish, especially because of variation in operating procedures between hatcheries. Captive environments relax natural selective pressures while exposing species to stressors that are unlike what is experienced in nature (Crates et al. 2023), and these environmental differences can result in phenotypic deviations from the wild state. Numerous studies on captive rearing have attempted to unravel the genetic effects of phenotype, only to find little or no genetic variation or effect of parentage across samples (Chittenden et al. 2010; Williamson et al. 2010; Hyvärinen and Rodewald 2013; Gavery et al. 2018). The root of this phenomenon could be linked to changes in transcription (Bull et al. 2022). For instance, rearing environment has been found to alter the expression of over 700 genes between hatchery and wild steelhead ( *O. mykiss* ), though these changes could not be linked to genetic drift or maternal effects (Christie et al. 2016). Instead, these results may point to transcriptional changes of epigenetic origin.

Epigenetic mechanisms may cause heritable changes to gene expression without a change in the DNA sequence (Best et al. 2018). The most commonly studied epigenetic mechanism, DNA methylation, is one modulator of gene expression that acts by regulating DNA transcription (Bird 2002; Ehrlich and Lacey 2013). In vertebrates, DNA methylation primarily occurs at CpG sites (Varriale 2014), a cytosine followed by a guanine in the DNA sequence. DNA methylation often affects transcription by either directly inhibiting transcription proteins from binding, or by recruiting co‐suppressor proteins that inhibit binding (Moore et al. 2013; Breiling and Lyko 2015). In contrast, hypermethylation of promoter regions can sometimes activate transcription (Smith et al. 2020), while gene body methylation may reduce or enhance gene expression (Ball et al. 2009; Uren Webster et al. 2018).

The influence of environment—especially early environment—on DNA methylation has also been well documented (Meaney and Szyf 2005; Angers et al. 2010; Flores et al. 2013), revealing that early rearing environment and subsequent patterns of DNA methylation may be influencing changes in gene expression. For instance, hypoxia (Kelly et al. 2020), environmental enrichment (Reiser et al. 2021), salinity (Heckwolf et al. 2020), disease (Sagonas et al. 2020), rearing temperature (Anastasiadi et al. 2017; Burgerhout et al. 2017), and diet (Panserat et al. 2017; Gavery et al. 2019) can all induce changes in DNA methylation. DNA methylation can also change relatively rapidly (Venney et al. 2023; Fox et al. 2025) and reversibly (Beemelmanns et al. 2021) in response to the environment, though other changes are irreversible (e.g., early developmental changes observed in Gavery et al. 2019). As DNA methylation is incredibly sensitive to environmental fluctuations, it provides a potential molecular mechanism contributing to phenotypic plasticity by modifying transcription in response to environmental change (Anastasiadi et al. 2021). Moreover, recent studies have found that epigenetic changes can persist within a lifetime and across multiple generations in fish indicating that at least some changes in DNA methylation can be intergenerationally stable (Hofmeister et al. 2017; Anastasiadi et al. 2021; Hu et al. 2021; Hirayama et al. 2024). Therefore, DNA methylation presents a potential mechanism towards consistent phenotypic differences between captive‐reared and wild fish. As a result, epigenetics has risen to the forefront of the hatchery dilemma, with studies attributing phenotypic abnormalities to interactions between the environment and the epigenome (Westley et al. 2013; Johnsson et al. 2014).

### 1.4 Parallelism Between Transcriptional and Epigenetic Effects of Captive Rearing

Captive rearing often has similar phenotypic effects across salmonids, and the effects of captive rearing on the fish epigenome have been reported for many species (Le Luyer et al. 2017; Gavery et al. 2018; Berbel‐Filho et al. 2020; Latorre et al. 2020; Leitwein et al. 2021; Habibi et al. 2024; Venney et al. 2025). Hatchery environments commonly affect the expression of immune response, developmental, and nervous system‐related genes. For instance, the upregulation of immune system developmental genes has been observed (Wellband et al. 2021), as well as down‐regulation of genes required in immune response (Leitwein et al. 2022). Regions related to coagulation and wound healing have been differentially expressed in salmonids as a result of captive rearing (Christie et al. 2016), along with those related to circadian rhythm (Leitwein et al. 2022) and metabolism (Christie et al. 2016). DNA methylation changes have also been documented at developmental genes associated with tissue and cell differentiation (Nilsson et al. 2021), liver development (Gavery et al. 2019), and the growth and development of skeletal muscles (Wellband et al. 2021), and they can impact SOX family transcription factors essential for sex‐determination (Rodriguez Barreto et al. 2019). In the nervous system, genes required for nervous system development (Rodriguez Barreto et al. 2019; Wellband et al. 2021), cell signaling (Rodriguez Barreto et al. 2019), neuromuscular communication (Le Luyer et al. 2017), and neurological regulation (Le Luyer et al. 2017; Wellband et al. 2021) have been differentially methylated in captive‐reared salmonids. Altered methylation states have also been discovered for genes involved in metabolism and immunity (Nilsson et al. 2021), stress and aggression response (Rodriguez Barreto et al. 2019), and homeostasis and osmoregulation (Le Luyer et al. 2017) in hatchery salmonids. Thus, differences in gene expression and DNA methylation affect genes across similar functional groups, and many of these functional groups are broadly consistent with the phenotypic effects of captive rearing discussed above. However, direct mechanistic links across these effects remain to be established in many cases. Regardless, while variation in epigenomic or transcriptomic responses to captivity could be due to captive rearing protocol, interspecies differences, or tissue‐specific gene expression patterns, the consistent implication of transcriptional and epigenomic changes emphasizes the importance of integrating epigenetic considerations into captive rearing.

**FIGURE 1:**
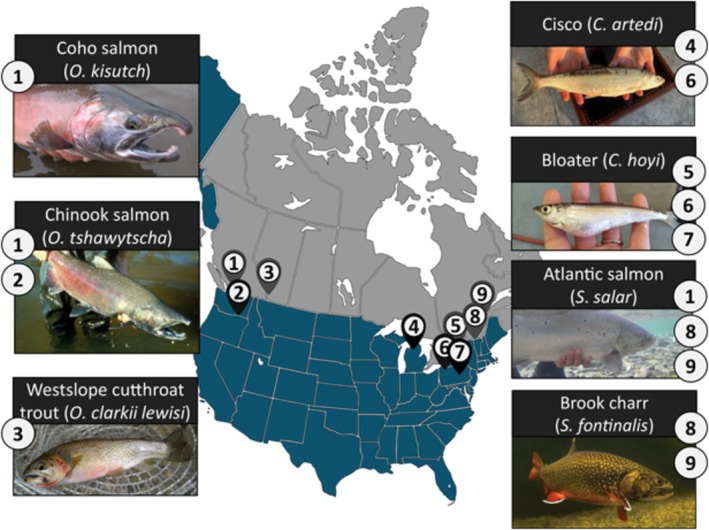
A selection of captive‐reared salmonids across Canada and the US studied by the co‐authors. Cisco photo credit: Nicole Watson, USGS. Bloater photo credit: Cory Brant, USGS.

As captive rearing becomes an increasingly applied tool for conservation, it is likewise increasingly important for scientists, hatchery managers, and governments to comprehensively understand and mitigate the negative effects of captive environments on fish. Accordingly, efforts are now being made to limit the phenotypic and/or epigenetic effects of captive rearing by modifying the hatchery environment and rearing protocols, with varying success (as reviewed in Anderson et al. 2020). Given our current understanding of the epigenetic effects of captive rearing, we ask: how can we utilize this knowledge to improve the success of conservation‐oriented captive rearing protocols? In this binational collaborative piece, we synthesize the knowledge and efforts of academics and government scientists involved in conservation‐oriented captive rearing (Figure 1) to highlight how and when epigenomics can be used to improve the captive rearing of salmonids. We discuss underreported phenotypic effects of captive rearing and their impact on salmonid post‐release survival and population fitness. We also consider the societal and scientific value of managing epigenetic changes in hatchery fish, and we assess modifications to hatchery environments for their economic and ecological value, as well as feasibility. We make recommendations to increase the fitness and survival of salmonids in the hatchery setting, as well as to utilize the capacity for plasticity to produce fish that are prepared for release into the wild. Finally, we propose integrating epigenetic considerations into the captive rearing protocols of salmonids to improve the future outlook of these programs. Our aim is to encourage harnessing epigenetic tools in captive rearing to improve conservation outcomes for salmonids worldwide.

## 2 Phenotypic Effects of Captive Rearing

**FIGURE 2:**
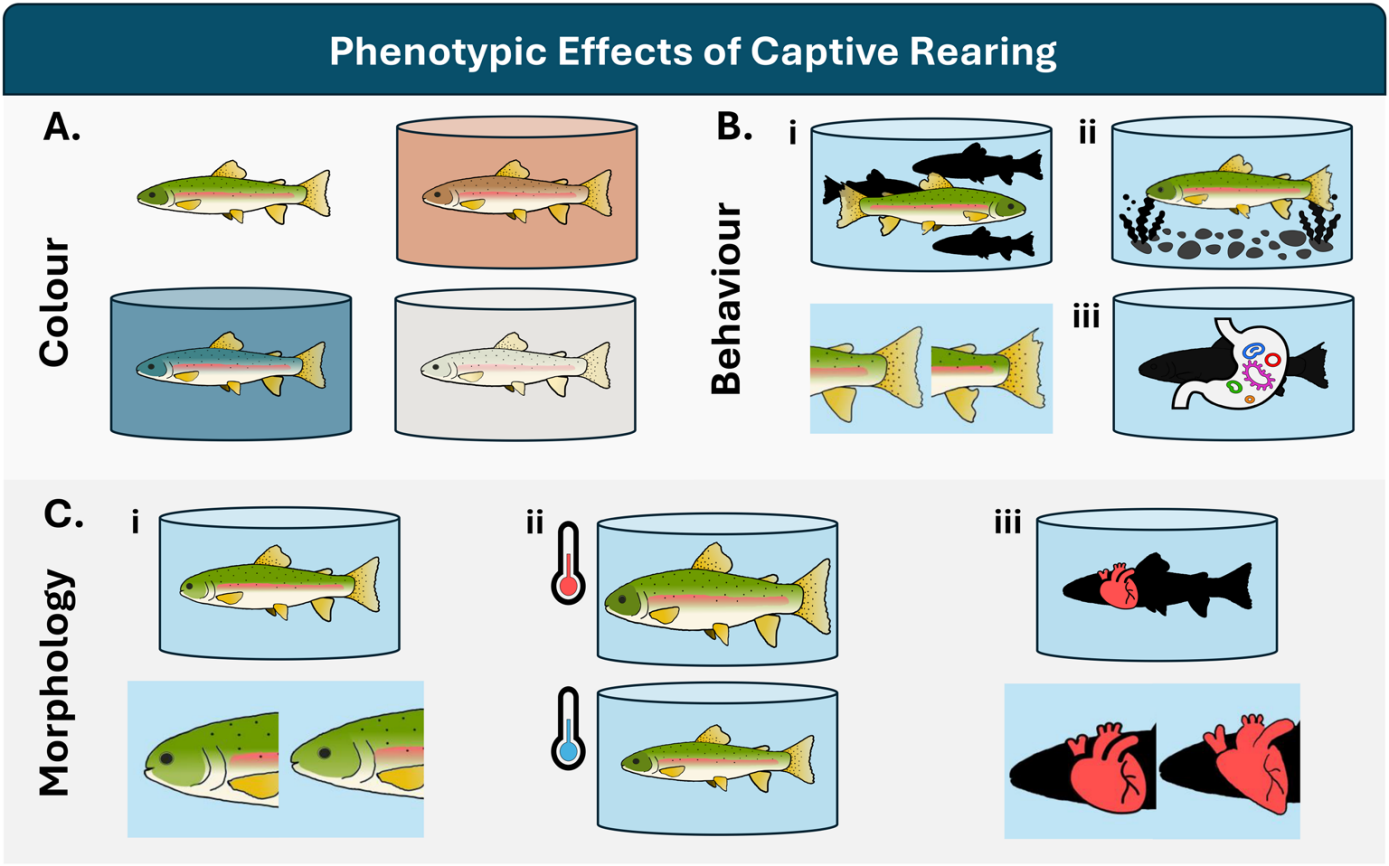
Some phenotypic effects of captive rearing on salmonids. (A) Color phenotypes mimic the rearing tank, resulting in abnormal shades and patterns relative to those found in nature. (B) Behavioral changes, indicated here as fin damage from nipping, could be linked to (i) high rearing density, (ii) lack of environmental enrichment, or (iii) altered gut microbiome. (C) (i) Pugheadedness, (ii) variation in size and condition factor, and (iii) changes to heart morphology can result from the captive environment.

Although fitness effects such as reduced reproductive success and survival are commonly reported for captive‐reared salmonids in nature (Araki et al. 2008; Bouchard 2021; Christie et al. 2012), many underreported or understudied hatchery‐induced phenotypes have implications for post‐release fitness (Figure 2).

### 2.1 Coloration

Captive environments often lead to altered external coloration in salmonids (Donnelly and Whoriskey 1991; Westley et al. 2013; Wang et al. 2017; Kasagi et al. 2020; reviewed in McLean 2021). Like other poikilotherms, salmonids have chromatophores (or color‐specific pigment cells) that are used to blend in with the surrounding environment and in interspecies communication (Fujii 2000; Sköld et al. 2016). In a hatchery facility, the environment is frequently barren, with external stimuli often limited to holding tank color. As a result, hatchery fish have been known to adopt their color and patterning from this environment—which varies between facilities—resulting in salmonids with unnatural pigmentation and limited patterning that reflects tank shade and design (Chittenden et al. 2010; Westley et al. 2013) (Figure 2A). Once released into the wild, a salmonid with non‐natural coloration may be at a selective disadvantage if it cannot adjust quickly to match the natural environment. For instance, fish with a lighter body tone may be more easily detectable by other animals, thus reducing their capacity to attain food and increasing their risk of predation. It is unclear to what extent coloration is plastic within a generation, though populations and species could differ in their capacity for plasticity in coloration (Sköld et al. 2016). Coloration and chromatophore function seem to be modulated by DNA methylation (Fang et al. 2026), though the link between epigenomics and coloration has only recently been investigated (Strowbridge et al. 2025). Because methylation state can change rapidly in response to environmental change (Venney et al. 2023; Fox et al. 2025), it is likely that coloration can also change fairly rapidly after environmental change. Given the role of coloration in sexual selection in salmonids (Craig and Foote 2001; Lehnert et al. 2016; Auld et al. 2019), an inability to alter coloration quickly could impact the reproductive success of fish if they do not adopt the appropriate spawning color. If coloration normalizes post‐release, then these effects may not be a concern, though short‐term fitness declines associated with altered fish coloration or increased predation could further affect post‐release survival.

### 2.2 Behavior

Behavioral changes in captive‐reared fish are one of the most commonly observed, reported, and studied phenotypic shifts to date (as reviewed in Johnsson et al. 2014). Studies have linked behavioral changes to high density rearing conditions (Fenderson and Carpenter 1971; Fleming and Gross 1993; Brockmark et al. 2010; Brockmark and Johnsson 2010) (Figure 2B(i)), environmental enrichment (Brown et al. 2003; Kihslinger and Nevitt 2006; Berbel‐Filho et al. 2020) (Figure 2B(ii)), differences in brain development (Kihslinger and Nevitt 2006), and domestication selection (Dender et al. 2018; Islam et al. 2020). Other possible explanations for altered behavior in captive salmonids include a lack of environmental stimuli conducive to complex neurological development and an absence of predators that reduces the need to develop predator avoidance and fear response (Jackson and Brown 2011). Moreover, domestication or habituation to human exposure may explain the bold and exploratory phenotype apparent in some studies (e.g., Vaz‐Serrano et al. 2011; Härkönen et al. 2014; Cogliati, Scanlan, et al. 2023), while excessive feeding and the consequential absence of intraspecific competition could be contributing to lower survival post‐release. Captive rearing protocols can also influence the rate at which fish learn to forage for natural prey after being reared on pellets (Rodewald et al. 2011).

#### 2.2.1 Behavior and the Microbiome

Captive rearing has been shown to affect the microbiome (Ramírez and Romero 2017; Minich et al. 2020; Kirchoff et al. 2022), likely due to diet differences between captive‐reared and wild fish (Lavoie et al. 2018; Yang et al. 2020) or disparities in bacteria between natural and hatchery water sources. Recently, work on Atlantic salmon has linked early rearing environment to significant differences in gut bacterial community structure between hatchery and wild fish (Lavoie et al. 2021). Researchers found that the gut microbiome was more affected by early rearing environment than diet or genotype and concluded that hatchery salmon possess a variable and immature microbial community structure, as well as a reduced ability to recruit key bacterial commensals in the gut (Lavoie et al. 2021).

The influence of the gut microbiome on vertebrate behavior is also becoming increasingly well known (Davidson et al. 2018; Sarkar et al. 2020) (Figure 2B(iii)). It has been suggested that behavioral plasticity may be moderated by the gut microbiome through the gut‐brain axis, whereby changes to the gut microbiome affect communication between the hypothalamus and hormone modulating glands (Davidson et al. 2020; Morais et al. 2021). If the gut microbiome is altered by captive rearing and influences behavior, then the behavioral effects commonly reported in hatchery‐reared fish may be partially due to the microbiome. Evidence for links between the microbiome and epigenome is limited, but these studies reinforce the need to further investigate potential epigenetic links between environment, microbiome, and behavior, which could be harnessed to improve captive rearing approaches (Consuegra et al. 2023).

### 2.3 Morphology

Captive rearing can also affect salmonid morphology. For example, captive‐reared bloater ( *C. hoyi* ) display shorter dorsal fins, smaller eyes, and shallower bodies compared to wild fish (Honsey, Kao, et al. 2024). In cisco ( *C. artedi* ), these alterations include deformed gill rakers, shallower bodies, smaller eyes, and varying levels of pugheadedness—irregular compression of the snout or forehead (Figure 2C(i); Honsey, Anweiler, et al. 2024). Pugheadedness has also been reported in captive reared Atlantic salmon (Jawad et al. 2014) and observed in captive westslope cutthroat trout (T. Attfield, personal observation). In rainbow trout and Atlantic salmon, differences in heart and valve morphologies between hatchery and wild fish have also been reported (Poppe et al. 2003) (Figure 2C(iii)). These deformities may affect swimming and migration capacity (Lijalad and Powell 2009), reduce gas exchange rate and cardiac function (Poppe et al. 2003; Lijalad and Powell 2009), impact filter feeding action (Amundsen et al. 2004; Noble et al. 2012; Honsey, Anweiler, et al. 2024), lead to stunted growth (Poppe et al. 2003), and increase mortality (Branson and Turnbull 2008). Further research into these deformities, their molecular basis, their effects on fitness, and their potential heritability is warranted to better understand how these trait variations might impact wild populations through interactions with captive‐reared fish. These traits may result from factors that are easily manipulated in hatcheries, such as rearing temperature (Figure 2C(ii)) or density, so further investigation into these effects may reveal simple mitigation strategies.

### 2.4 Life History Effects

Alterations to life history strategies are another concern for hatchery salmonids. Broadly, life history strategies encompass age‐specific traits and timing of milestones related to growth, reproduction, and survival (Winemiller 2005). In salmonids, these may include actions associated with migration, smoltification, spawning, and developmental strategies. Rearing environment has been found to impact these traits in salmonids, specifically regarding precocious male development and migratory strategy (Chittenden et al. 2010; Hyvärinen and Rodewald 2013) as well as in spawning site selection, nest quality, and redd abandonment (Schroder et al. 2008). Some explanations for these differences include reduced swimming and migration speed due to higher fat accumulation in the bodies and heart of hatchery salmon (Poppe et al. 2003; Chittenden et al. 2010; Molversmyr et al. 2022) or various changes to morphology (Poppe et al. 2003; Honsey, Anweiler, et al. 2024). Some of these life history differences could be related to hatchery‐induced behavioral changes, though it is difficult to pinpoint a single cause of altered life history strategy.

At least one difference in life history strategies, the process of homing, has a plausible connection with epigenetic variation. Although it is not fully understood how salmon imprint on their early environment and find their way back to their spawning grounds, it is agreed that at least part of this process relies on olfaction (Cooper and Scholz 1976; Stabell 1984; Dittman and Quinn 1996). At the genome level, olfactory gene expression has been found to occur transiently, whereby gene expression changes during smoltification and during the migration back to natal spawning grounds (Dukes et al. 2004; Ueda et al. 2016; Bellinger et al. 2022). Specifically, increased expression of the memory gene *NR1* has been found to occur during these migrations (Ueda et al. 2016). As this gene has been linked to memory of olfactory cues, its increased expression during smolt and spawning migration may facilitate the formation and retrieval of these olfactory memories in salmonids. Hatchery fish often show a higher tendency to disperse compared to wild fish—a pattern reviewed in Lamarins et al. (2024). This is likely because environmental imprinting occurs in the alevin or embryo stage in salmon life histories (Quinn et al. 2006; Bett et al. 2016), which often corresponds to the pre‐release period of hatchery fish. Still, morphological differences observed between olfactory systems in hatchery and wild sockeye salmon (Ward et al. 2024), as well as epigenetic changes to olfactory genes in response to captive environments (Rodriguez Barreto et al. 2019), may point to an epigenetic mechanism driving the differences in homing and dispersal in captive reared salmonids. Further study of the epigenetic impacts of captive rearing on olfactory epigenetics could explain the loss of homing behavior in some captive‐reared salmonids.

## 3 Current Efforts to Minimize the Phenotypic and Epigenetic Effects of Captive Rearing

### 3.1 Modifying the Hatchery Environment

The effects of captive rearing are often minimized by replicating the natural environment (Koch et al. 2023). Current attempts to replicate the natural environment have included adjusting water temperature to reflect natural rearing conditions (Spangenberg et al. 2014; Harstad et al. 2018); enriching tanks with gravel, astroturf, or hiding spots (Brown et al. 2003; Kihslinger and Nevitt 2006; Hyvärinen and Rodewald 2013; Reiser et al. 2019); and building simulated nests during egg development (Reiser et al. 2019). Semi‐natural environments are also common in captive rearing, including ponds, raceways (Brignon et al. 2012), and simulated streams (Schroder et al. 2008, 2010) that are exposed to the elements. Research has shown that the unpredictable conditions in these systems increase post‐release fitness (Braithwaite and Salvanes 2005; Beckman et al. 2017), and suggestions have been made to introduce variability in hatcheries specifically to prepare fish for the ever‐changing natural environment (Johnsson et al. 2014). Although many hatcheries are unable to switch to semi‐natural systems due to space constraints or cost, managers interested in stocking for conservation may be more eager to adopt these approaches if they result in phenotypically “wild” fish.

### 3.2 Optimizing Rearing Protocols

#### 3.2.1 Incubation and Early Rearing Protocols

Modifications may also be made to hatchery protocols to reduce their unintended phenotypic and epigenetic effects. This could include rearing density reduction (Alanärä and Brännäs 1996; Brockmark et al. 2007), modifying feeding protocols, or reducing residence time. Densely reared eggs will suffer some degree of hypoxia during incubation (Wood et al. 2020), which can result in impeded growth, development, and survival, potentially due to epigenetic changes (Beemelmanns et al. 2021). Therefore, reducing egg density or rearing eggs in seminatural incubators (Bamberger 2009) could be considered to increase egg to fry survival. Supplementation with probiotics can be used to improve the microbiome of captive reared fish (Akbari Nargesi et al. 2020; Sumon et al. 2022), while feeding or training fish with live prey may also help to increase fitness (Brown et al. 2003). Alternatively, it might be beneficial to alter the residence time of salmonids in a hatchery environment to increase post‐release survival and fitness (Milot et al. 2013). Although most epigenetic modifications occur during early rearing, research suggests that changes in methylation continue up to a year in the hatchery and during gametogenesis (Gavery et al. 2019). However, the persistence of these plastic responses to rearing environment can also be used to improve captive rearing. The temporary release of hatchery‐reared juveniles into nature 1 or 2 years before recapture led to a two‐fold increase in the survival of their offspring in the wild (Evans et al. 2014). The effects of captive rearing clearly persist within a generation after release into the wild (Evans et al. 2014; Leitwein et al. 2021; Nuetzel et al. 2023; Dayan et al. 2024; Venney et al. 2025), so integrated broodstocks may not be enough to curb the fitness effects of captivity. Consequently, it may be better to release salmonids prior to the development of later‐stage—and potentially irreversible—epigenetic modifications, as the plastic epigenome of early‐stage salmonids may allow for better acclimation to the natural environment and restoration of the wild epigenome.

#### 3.2.2 Gamete Collections and Spawning Protocols

Wild‐caught (Evans et al. 2014; Fast et al. 2015) or integrated broodstocks (Koch et al. 2023) may be utilized to maintain the wild epigenome in the hatchery, though transporting sexually mature fish may result in altered offspring development, since epigenetic changes can be induced during gametogenesis (Labbé et al. 2017). Environmental disturbances or acclimation to the captive environment during gamete maturation could explain some phenotypic effects induced by captive rearing. Indeed, manipulating thermal regime during adult sexual maturation led to stable epigenetic differences in juvenile brook charr ( *Salvelinus fontinalis* ) regardless of their own thermal regime (Venney et al. 2022). Logistically, it is often preferable to transport adult fish into the hatchery for captive breeding (e.g., to enrich broodstocks), but the effects of the degree of handling, length of transportation, or captive environmental conditions could influence the extent of epigenetic changes. Some rearing procedures can avoid transferring adults into captivity by direct harvesting from natural redds (Berejikian et al. 2011), or relying on gamete collections in the wild followed by fertilizations in the hatchery.

Delaying the fertilization of collected eggs and milt can affect egg survival (Eide and Barnes 2018). Methods of storing gametes prior to fertilization can also impact survival and gamete DNA methylation (Ciereszko et al. 2020; Cheng et al. 2024). Cryopreservation of milt, though intended to maintain the genetic diversity of salmon, likely influences the epigenome (Depincé et al. 2020; El Kamouh et al. 2023). In an experiment that measured epigenetic signatures in milt following cold storage and cryopreservation, DNA methylation in Atlantic salmon milt increased after only 1 day of cold storage at 2°C (Narud et al. 2023). Although 1 day of cryopreservation did not significantly impact milt DNA methylation in this study (Narud et al. 2023), longer‐term studies have shown that cryopreservation affects the sperm epigenome (Wang et al. 2022; Lee et al. 2023). Gamete storage and spawning protocols are likely to have more considerable effects on captive‐reared offspring, yet it remains unclear whether parental captivity or in‐field gamete collections could improve conservation outcomes, such as by reducing epigenome changes or increasing hatch and survival rates.

## 4 The Value of Minimizing the Epigenetic Effects of Captive Rearing

Given the amount of effort required to modify the hatchery environment and minimize the epigenetic and phenotypic effects of captive rearing, under what scenarios are the time and financial investments worthwhile?

### 4.1 Societal Perspectives

From a conservation and economic perspective, reducing the phenotypic effects of captive rearing for put‐and‐take stocking of isolated water bodies might not be worthwhile. However, anglers generally want to catch and eat wild‐looking fish with typical coloration patterns and flesh color (Alfnes et al. 2006; Colihueque 2010). Although some anglers prefer catching numerous fish of harvestable size, others desire catching fewer, larger trophy fish (Beardmore et al. 2015). Mature hatchery salmonids can have smaller bodies than their wild counterparts (Schroder et al. 2010; de Mestral et al. 2013; Wiper et al. 2014), as well as homogeneity of body size at the time of release, limiting the number of true trophy‐sized fish accessible to anglers. Some studies have found hatchery fish to have greater mass than wild conspecifics (Hill et al. 2006; Chittenden et al. 2010; Cogliati, Noakes, et al. 2023), though this has been associated with higher fatty tissue (Chittenden et al. 2010; Cogliati, Noakes, et al. 2023) rather than lean mass. However, wild fish are currently trending towards smaller body sizes and younger age classes, which may negate this finding as stocks evolve (Oke et al. 2020). Thus, while put‐and‐take fisheries may not require extensive modification of the captive environment, angler satisfaction may be higher if there is greater effort to produce wild‐looking fish.

Salmonids also possess significant cultural value for Indigenous Communities in Canada and the United States (Trushenski et al. 2018) and many species have served as a traditional food source for centuries (Berkes 1990; Reid et al. 2022). As such, hatcheries play a key role in maintaining Indigenous peoples' livelihoods by supplementing dwindling salmonid populations in historical basins (Bingham et al. 2021; Blanchet et al. 2022). To restore and preserve natural salmonid populations, it is essential to raise fish that are phenotypically similar to their wild counterparts so that their fitness remains unaffected (Fraser 2008). This is essential for the preservation of genetic diversity over time (Hindar et al. 1991; Ryman and Laikre 1991; Waples 1991), as well as for conserving local adaptations (Fraser 2008), both of which can ultimately influence traditional harvesting practices (Marin et al. 2017; Connoy et al. 2024; FiveCrows et al. 2025). As there is mounting support for the idea that fitness changes could be caused by epigenetic differences between hatchery and wild fish (Le Luyer et al. 2017; Wellband et al. 2021; Venney et al. 2023), efforts should be made in these watersheds to release epigenetically wild salmonids.

### 4.2 Scientific and Management Perspectives

**FIGURE 3:**
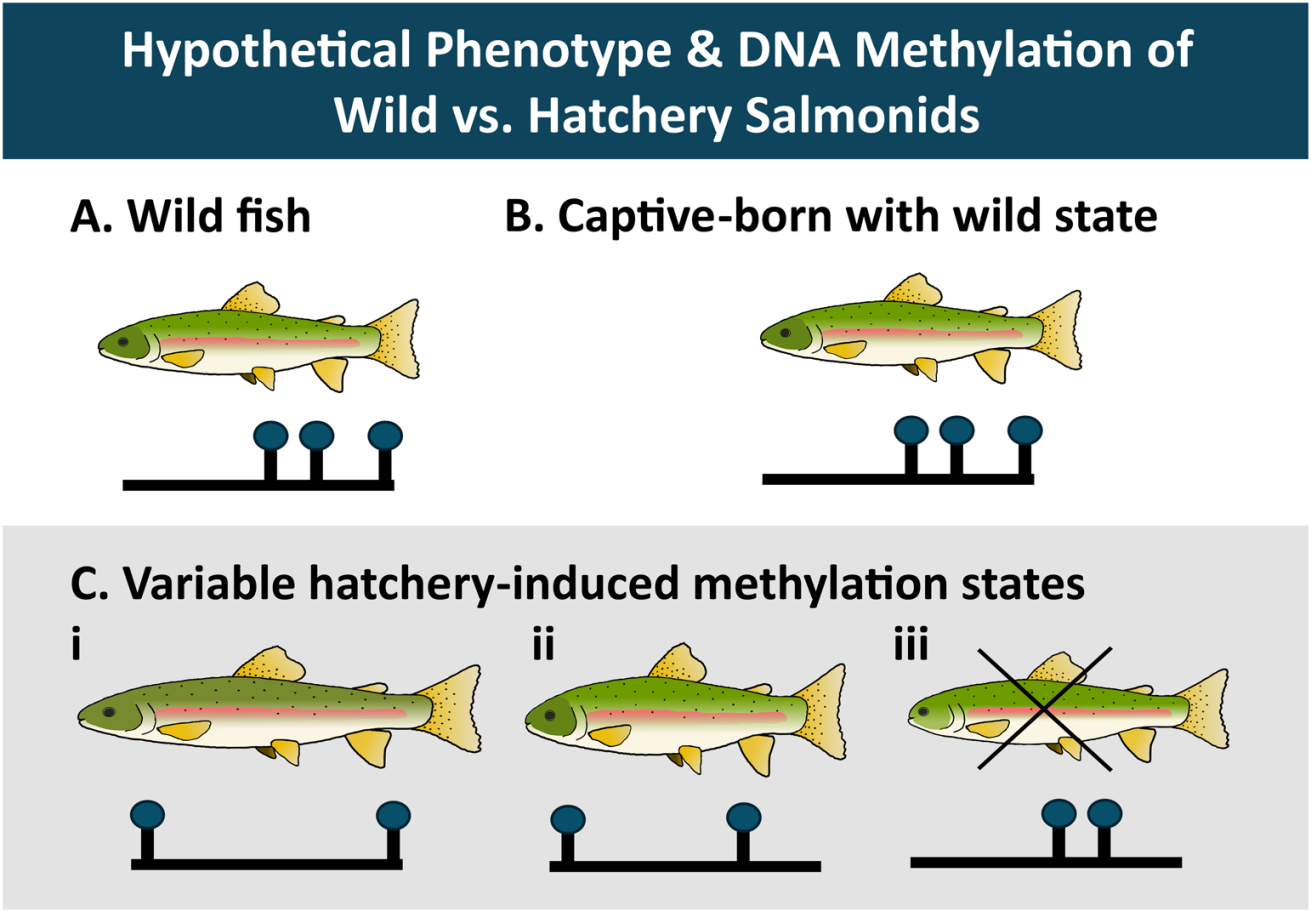
DNA methylation states, phenotype, and post‐release survival of captive salmonids. (A) A wild fish with wild methylation patterns. (B) Captive rearing conditions could result in wild‐like methylation, specifically if conditions mimic the natural environment. (C) Rearing conditions and their subsequent methylation patterns may bolster post‐release fitness such as through changes in color (i) or size (i and ii), but methylation that results in abnormal phenotypes, like pugheadedness (iii), could impact post‐release survival.

Minimizing the phenotypic and epigenetic effects of captive rearing is most crucial when the goal is to conserve while supplementing wild populations. If a hatchery‐induced phenotype is detrimental to salmonid fitness in the wild, efforts should be made to minimize the production of these maladaptive phenotypes in the hatchery (Figure 3). An additional scientific goal of captive rearing should be to produce fish that are prepared for the natural environment they will encounter (Figure 3B,C). Selection is generally relaxed in the hatchery environment, leading to altered transcriptional and epigenetic states which may confer an advantage in the hatchery but are generally detrimental in nature (Araki et al. 2008). Releasing maladapted fish with low probability of survival or reproductive success into the wild would be of little benefit to wild populations or conservation efforts, and can actually negatively affect population productivity (O'Sullivan et al. 2020). To improve the societal benefit and economic value of salmonid supplementation programs, the survival rate and reproductive success of captive‐reared fishes need to be improved (Larsson et al. 2011).

#### 4.2.1 Heritability

Given that the epigenetic effects of captive rearing could persist across generations, captive rearing could lead to multi‐generational effects on wild populations. For changes in methylation to be heritable across generations, they must occur in germ cells. Several studies have reported the persistence and heritability of the epigenetic effects of captive rearing. Some have documented persistence of epigenetic markers over a year after release (Leitwein et al. 2021; Venney et al. 2025) or have observed epigenetic (Wellband et al. 2021; Venney et al. 2025) and phenotypic (Wellband et al. 2021) changes into the F1 generation. Although the effects of captive rearing have not been observed beyond the F1 generation, efforts should be made to correct these phenotypes to prevent negative fitness effects in wild populations.

## 5 Fitter Fish: Wild or More Variable?

Should we seek to produce “wilder” fish, or produce fish that can withstand the anthropogenic threats of tomorrow? Can we produce fish that are better suited for a climate change‐afflicted future simply by exposing them to increased rearing temperatures or acute thermal stress? Could rearing in low‐oxygen conditions help salmonids tolerate oxygen‐starved waterbodies? It may be possible to use the time a fish spends in captivity to improve its survival in the wild, especially if the characteristics and stressors of the post‐release environment are known or predictable.

**FIGURE 4:**
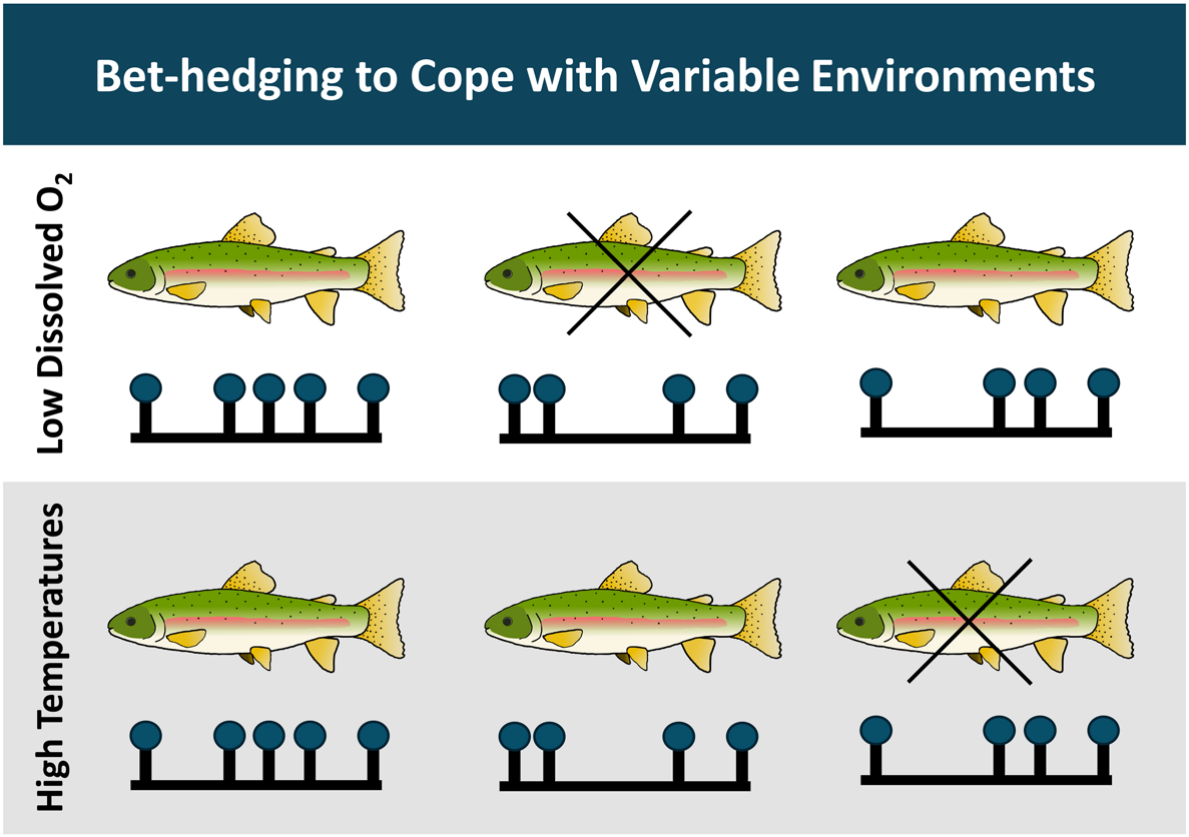
Rearing conditions that encourage epigenetic variability (i.e., bet‐hedging) could produce fish better adapted to changing environmental conditions including increased temperatures or oxygen deprived waters.

When the post‐release environment is less predictable or uncharacterized, epigenetics could be harnessed to create fish that are more capable of withstanding variable or unpredictable environments. Heritable bet‐hedging describes the process of increasing heritable epigenetic and phenotypic variation among offspring, leading to a reduction in individual fitness, but a greater likelihood that certain offspring will manage to survive and further acclimate to the environment (Figure 4). It has been suggested that increasing interindividual variation among offspring, or increasing offspring capacity for plasticity, can help species combat rapidly changing environments (O'Dea et al. 2016; Angers et al. 2020). We may be able to use this capacity for plasticity to epigenetically “buffer” fish to better survive in current and future environments. In practice, this could entail exposing hatchery fish to varying rearing temperatures throughout development, or rearing fish in the variability of an outdoor environment. Mechanistically, many hatcheries can alter rearing temperature, making this research feasible in current hatchery frameworks.

However, this method of epigenetic buffering is heavily dependent on the amount of epigenetic variation present among hatchery fish and their offspring. Utilizing epigenetic buffering would first require quantifying epigenetic variation in salmonids to understand if hatchery salmonids even possess enough variation for successful buffering to occur. There is a greater probability that epigenetic buffering could be useful if offspring already have high epigenetic variation or capacity for plasticity, or if offspring can be produced to maximize epigenetic and phenotypic variation. Therefore, if we were to compare the amount of variation between hatchery and wild salmonids and discover that wild salmonids have greater epigenetic variation, this might encourage managers to use native broodstock to maintain a diversified epigenome, or direct research towards methods of maintaining high epigenetic and phenotypic variation in hatchery fish. Understanding how to manipulate epigenetic theories such as heritable bet‐hedging could allow us to improve the fitness of first‐generation hatchery fish. This would necessitate introducing variable phenotypes into a population in the hope that they would provide a selective advantage that encourages the persistence of at least some individuals.

## 6 Epigenetic Biomarkers and Post‐Release Monitoring

Another potential use of epigenomics for fish conservation is for post‐release monitoring through epigenetic biomarkers (Anastasiadi and Beemelmanns 2023). Epigenetic biomarkers have already been developed in zebrafish ( *Danio rerio* ) to identify early‐life exposure to high temperatures (Valdivieso et al. 2023), but they have not yet been developed in salmonids. Still, parallel epigenetic signatures of domestication have been found in different populations of coho salmon ( *Oncorhynchus kisutch* ) (Le Luyer et al. 2017), and across tissues in the liver and sperm of hatchery‐reared Atlantic salmon (Wellband et al. 2021), highlighting the potential for future biomarker development to differentiate wild and captive‐reared fish. Biomarkers can also inform on the age of a fish, such as through the development of epigenetic clocks (Lu et al. 2023). These epigenetic biomarkers and clocks could provide a substantial amount and diversity of information using a single non‐lethal sample, such as blood or fin clip samples. Cross‐species utility should be analyzed in salmonids to increase the efficacy of epigenetic biomarkers in captive rearing and post‐release monitoring.

## 7 Conclusions and Future Directions

The negative phenotypic and fitness effects of traditional captive rearing methods have become increasingly apparent, though much is still unknown about the potential role of epigenetic variation in driving these maladaptive phenotypes. However, two crucial circumstances must be met before epigenomics can be applied to improve captive rearing and hatchery protocols on a broad scale. First, we must build consensus on the role and importance of epigenetic variation in determining hatchery phenotypes, and the potential to mitigate these effects through changes in production practices. Second, we must demonstrate tangible benefits of reversing these epigenetic deviations such that hatchery production can be tailored both to meet management objectives and to minimize impacts on wild fish. If targeting the epigenetic effects of captive rearing can reduce risks to fitness and productivity of natural populations, it may be easier to justify incorporating changes in hatchery protocol that maintain the wild epigenome. If these modifications also bolster conservation efforts by minimizing supplementation effort required to support populations, less hatchery resources would be consumed, thus reducing the cost of production. As a result, taking epigenetics into consideration when designing hatchery protocols could aid conservation efforts and improve cost‐effectiveness if the above possibilities hold true. Therefore, the collective consideration of epigenetic effects between hatchery managers, scientists, and governments may be an important component of conserving salmonid populations for ongoing cultural, environmental, and economic benefits for future generations.

## Funding

This study was supported by the U.S. Geological Survey (G25AC00208‐00) and Natural Sciences and Engineering Research Council of Canada (RGPIN‐2025‐04324).

## Conflicts of Interest

The authors declare no conflicts of interest.

## Data Availability

Data sharing not applicable to this article as no datasets were generated or analyzed during the current study.
